# Continuous Invasion by Respiratory Viruses Observed in Rural Households During a Respiratory Syncytial Virus Seasonal Outbreak in Coastal Kenya

**DOI:** 10.1093/cid/ciy313

**Published:** 2018-04-16

**Authors:** Patrick K Munywoki, Dorothy C Koech, Charles N Agoti, Patricia A Cane, Graham F Medley, D James Nokes

**Affiliations:** 1 Epidemiology and Demography Department, Kenya Medical Research Institute–Wellcome Trust Research Programme, Centre for Geographic Medicine Research–Coast; 2 Department of Nursing and Public Health, Pwani University, Kilifi, Kenya; 3 High Containment Microbiology, Public Health England, Salisbury; 4 Department of Global Health and Development, London School of Hygiene and Tropical Medicine, University of London; 5 School of Life Sciences and Zeeman Institute for Systems Biology and Infectious Disease Epidemiology Research, University of Warwick, Coventry, United Kingdom

**Keywords:** respiratory viruses, transmission, household, developing countries, Kenya

## Abstract

**Background:**

Households are high-intensity close-contact environments favorable for transmission of respiratory viruses, yet little is known for low-income settings.

**Methods:**

Active surveillance was completed on 47 households in rural coastal Kenya over 6 months during a respiratory syncytial virus (RSV) season. Nasopharyngeal swabs (NPSs) were taken from 483 household members twice weekly irrespective of symptoms. Using molecular diagnostics, NPSs from 6 households were screened for 15 respiratory viruses and the remainder of households only for the most frequent viruses observed: rhinovirus (RV), human coronavirus (HCoV; comprising strains 229E, OC43, and NL63), adenovirus (AdV), and RSV (A and B).

**Results:**

Of 16928 NPSs tested for the common viruses, 4259 (25.2%) were positive for ≥1 target; 596 (13.8%) had coinfections. Detection frequencies were 10.5% RV (1780), 7.5% HCoV (1274), 7.3% AdV (1232), and 3.2% RSV (537). On average, each household and individual had 6 and 3 different viruses detected over the study period, respectively. Rhinovirus and HCoV were detected in all the 47 households while AdV and RSV were detected in 45 (95.7%) and 40 (85.1%) households, respectively. The individual risk of infection over the 6-month period was 93.4%, 80.1%, 71.6%, 61.5%, and 37.1% for any virus, RV, HCoV, AdV, and RSV, respectively. NPSs collected during symptomatic days and from younger age groups had higher prevalence of virus detection relative to respective counterparts. RSV was underrepresented in households relative to hospital admission data.

**Conclusions:**

In this household setting, respiratory virus infections and associated illness are ubiquitous. Future studies should address the health and economic implications of these observations.

## Teaser

This is the first household study of multiple respiratory viruses in a low-income setting that used intensive sampling regardless of symptoms. There was a remarkably high frequency of virus infection and illness, demonstrating the opportunity for complex interactions.

The current understanding of respiratory virus epidemiology arises mainly from analysis of specimens collected from individuals seeking care at a hospital or health facility, usually focusing on a single virus. This approach cannot provide a complete description of viruses in circulation in the community. A proportion of the infections will be asymptomatic or not severe enough to require medical attention, and, respiratory virus infections are typically of short duration. Hence, a full ecological/epidemiological description requires frequent sampling of individuals in a population regardless of symptoms, which is rarely undertaken. As a result, our understanding of seasonality, persistence patterns, and transmission dynamics of most respiratory viruses at the community level remains uncertain. Increased sensitivity and range of pathogens detectable by molecular diagnostics over traditional methods (culture isolation or antigen detection) [1–3] enable enhanced studies of a wide range of respiratory viruses in otherwise healthy populations.

The present study involved respiratory virus screen of >16000 respiratory specimens that were collected from members of a rural coastal community in Kenya. The specimens were collected through household-based active surveillance for 6 months. Deep nasopharyngeal swabs (NPSs) were collected from all household members irrespective of symptoms. The intensive surveillance provided detailed infection data that allowed comprehensive investigation of the circulation of the respiratory viruses in the community. Previous reports have described the data on respiratory syncytial virus (RSV) in detail [4–7], and here we present data on a wide range of respiratory viruses.

## MATERIALS AND METHODS

### Data

The current analysis is of data from a household cohort study undertaken in rural coastal Kenya within the Kilifi Health and Demographic Surveillance System [8]. The study period spanned from 8 December 2009 to 5 June 2010. The study design and the details of field operations have been previously described [4–7]. Identifying who infects the infant with RSV in the household was the primary objective of the study [6]. Households were eligible if they had an infant born since the end of the previous RSV epidemic in the study location and at least 1 older sibling (aged <13 years). The study period spanned 1 complete RSV season [6]. Deep NPS collections were requested from all household members irrespective of symptoms, once weekly in the first 4 weeks and subsequently twice weekly for the remainder of the study period. Retention of households and individuals in the study was >80% [6].

### Respiratory Virus Screening Using Multiplex Real-Time Polymerase Chain Reaction

By multiplex real-time polymerase chain reaction assay, NPS collections from 6 households were screened for 15 respiratory virus targets as previously described [6, 9]. These households were selected to represent various household sizes (range, 4–37 members). The full assay targets were RSV A and B, rhinovirus (RV), human coronavirus (HCoV-OC43, HCoV-NL63, and HCoV-229E), adenovirus (AdV), parainfluenza virus (PIV types 1–4), influenza (types A, B, and C), and human metapneumovirus (HMPV). For the remainder of the NPS collections (from 41 households), screening was limited to the viruses (or virus groups) found most prevalent in the full screen, namely, RV, HCoV (OC43, NL63, 229E), AdV, and RSV (A and B). A specimen with a cycle threshold value of ≤35.0 for a specific virus target was considered positive. Targets with a detection rate of >5% were considered prevalent and constitute targets taken forward for screening of all the NPS collections.

### Statistical Analysis

Data analyses were done with Stata version 13.1 software (StataCorp, College Station, Texas). Appropriate statistical tests were used that included the Student *t* test, χ^2^ test, and Fisher exact test. Week-delimited data on virus detections were plotted to show the temporal distributions and co-circulation at sampling, individual, and household level. Overall prevalence of the detected respiratory pathogens in households, individuals, and samples is also shown. The crude household and individual attack rates (defined as the household and individual risk of infection over the 6 months, respectively) were stratified by age, symptom status, household size, and gender.

### Ethical Considerations

An informed written consent was obtained from all the study participants or their parents/guardian. Ethical approval for the study was provided by the Kenya Medical Research Institute Scientific and Ethical Review Committee in Kenya and the University of Warwick Biomedical Research Ethical Committee in the United Kingdom.

## RESULTS

### Baseline Characteristics

The median occupancy in the 47 households was 8 members (range, 4–37). The average age of the members in each household at the start of sampling was 15.5 (95% confidence interval, 13.2–17.9) years. The baseline characteristics of the 6 households that were screened for all the 15 respiratory targets compared to the 41 households whose samples were tested for only the most prevalent respiratory viruses were similar, apart from the latter having a higher proportion of school-going children (25.3% vs 36.6%, χ^2^*P* value = .049; Table 1). Overall, data from the 47 households with 483 participants are presented. Ten participants who were never sampled were excluded from the subsequent analysis. A total of 16928 samples collected were tested: 2844 samples from the 6 households (80 individuals) with full respiratory virus screen and 14084 samples from the remaining 41 households (403 individuals) with select respiratory virus screen.

**Table 1.** Baseline Characteristics of the Households and Individuals With Select and Full Respiratory Virus Screening

Characteristic	Full Screen (83 Participants, 6 Households)	Select Screen (410 Participants, 41 Households)	*P* Value
Household size, median (interquartile range)	10.5 (5–15)	8 (7–11)	.8602
School-going children	21 (25.3)	150 (36.6)	.049
Male sex	31 (37.4)	190 (46.3)	.133
Number of specimens per person			
0	7 (1.7)	3 (3.6)	.437
1–9	34 (8.3)	5 (6.0)	
10–19	18 (4.4)	5 (6.0)	
20–29	38 (9.3)	8 (9.6)	
30–39	100 (24.4)	14 (16.9)	
40–44	140 (34.2)	36 (43.4)	
45–50	73 (17.8)	12 (14.5)	
Age group, y^a^			
<1	10 (12.1)	45 (11.0)	.467
1–4	16 (19.3)	66 (16.1)	
5–14	24 (28.9)	141 (34.4)	
15–39	22 (26.5)	125 (30.5)	
≥40	11 (13.3)	33 (8.1)	

Data are presented as No. (%) unless otherwise indicated.

^a^Age at start of sampling.

### Viruses Detected From Full Respiratory Virus Screen

One or more of the 15 respiratory viruses were detected in 864 of 2844 (30.4%) of the NPS collections, of which 714 (82.6%), 126 (14.6%), 19 (2.2%), 4 (0.5%), and 1 (0.1%) had 1, 2, 3, 4, and 5 viruses, respectively, (co-)detected. The proportion of samples that were virus positive was higher for specimens collected while the individual had symptoms compared with specimens collected during asymptomatic periods (52.0% [275/529] vs 25.4% [589/2315], respectively; χ^2^*P* < .0001). Those NPS specimens with multiple virus detections had increased frequency of symptoms over single infections (39.3% [59/150] vs 30.3% [216/714]; *P* = .03). The details of the number of samples that were positive for the respective targets are provided in Table 2. Of the 2844 NPS collections screened, the number positive, by pathogen, was 302 (10.6%) for RV, 270 (9.5%) for AdV, 217 (7.6%) for HCoV, 151 (5.3%) for RSV, 63 (2.2%) for PIV, 13 (0.5%) HMPV, and 11 (0.4%) for influenza. Of the virus positives, the corresponding number of samples collected from individuals with symptoms, by pathogen, were 103 (34.1%), 93 (34.4%), 68 (31.3%), 50 (33.1%), 18 (28.6%), 2 (15.5%), and 6 (54.5%).

**Table 2.** Respiratory Virus Detections in Households, Participants, and Nasopharyngeal Swab Collections, by Screening Strategy

Description	Full Respiratory Virus Screen						Select Respiratory Virus Screen					
	Household (n = 6)		Participants^a^ (n = 80)		Samples (n = 2844)		Household (n = 47)		Participants^a^ (n = 483)		Samples (n = 16918)	
Any virus detected (all)	6	(100.0)	75	(93.8)	864	(30.4)	…	…	…	…	…	…
Any virus detected (select)	6	(100.0)	75	(93.8)	803	(28.2)	47	(100.0)	451	(93.4)	4259	(25.2)
Rhinovirus	6	(100.0)	62	(77.5)	302	(10.6)	47	(100.0)	387	(80.1)	1780	(10.5)
Adenovirus	6	(100.0)	57	(71.3)	270	(9.5)	45	(95.7)	297	(61.5)	1232	(7.3)
Human coronavirus	6	(100.0)	58	(72.5)	217	(7.6)	47	(100.0)	346	(71.6)	1274	(7.5)
OC43	5	(83.3)	45	(56.3)	116	(4.1)	44	(93.6)	215	(44.5)	651	(3.8)
NL63	4	(66.7)	35	(43.8)	95	(3.3)	33	(70.2)	163	(33.7)	418	(2.5)
229E	3	(50.0)	7	(8.8)	8	(0.3)	30	(63.8)	119	(24.6)	241	(1.4)
Respiratory syncytial virus	6	(100.0)	52	(65.0)	151	(5.3)	40	(85.1)	179	(37.1)	537	(3.2)
Group A	5	(83.3)	33	(41.3)	86	(3.0)	25	(53.2)	88	(18.2)	250	(1.5)
Group B	5	(83.3)	21	(26.3)	66	(2.3)	34	(72.3)	113	(23.4)	306	(1.8)
Parainfluenza virus	6	(100.0)	37	(46.3)	63	(2.2)	…	…	…	…	…	…
Type 1	4	(66.7)	6	(7.5)	6	(0.2)	…	…	…	…	…	…
Type 2	3	(50.0)	14	(17.5)	16	(0.6)	…	…	…	…	…	…
Type 3	5	(83.3)	19	(23.8)	30	(1.1)	…	…	…	…	…	…
Type 4	5	(83.3)	14	(17.5)	21	(0.7)	…	…	…	…	…	…
Human metapneumovirus	4	(66.7)	11	(13.8)	13	(0.5)	…	…	…	…	…	…
Influenza virus	4	(66.7)	8	(10.0)	11	(0.4)	…	…	…	…	…	…
Type A	3	(50.0)	5	(6.3)	7	(0.2)	…	…	…	…	…	…
Type B	1	(16.7)	2	(2.5)	2	(0.1)	…	…	…	…	…	…
Type C	2	(33.3)	5	(6.3)	6	(0.2)	…	…	…	…	…	…
Upper respiratory tract infection	6	(100.0)	63	(78.8)	529	(18.6)	47	(100.0)	403	(83.4)	3564	(21.1)

Data are presented as No. (%).

^a^Excludes 3 and 10 participants from full and select pathogen screening, respectively, who were never sampled.

**Figure 1.**
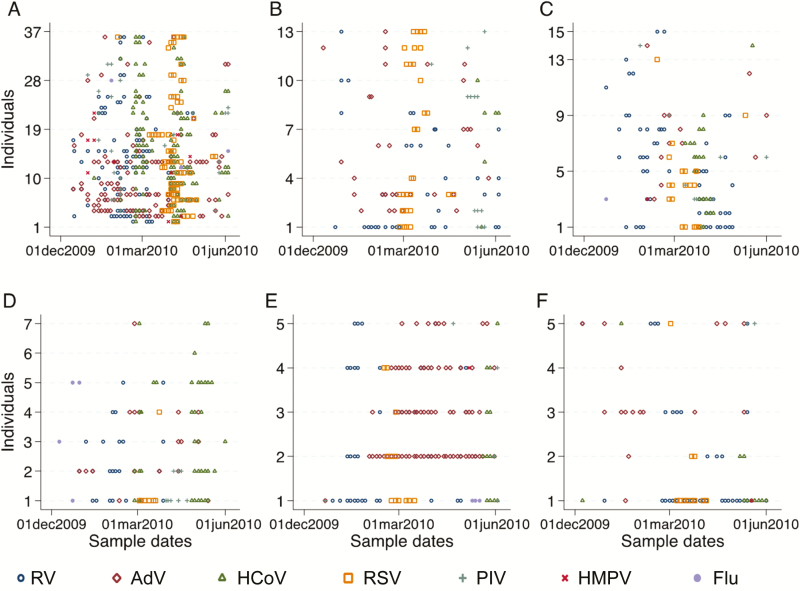
Temporal infection profile for the 6 households (*A–F*) showing positive samples for each member. Abbreviations: AdV, adenovirus; Flu, influenza; HCoV, human coronavirus; HMPV, human metapneumovirus; PIV, parainfluenza virus; RSV, respiratory syncytial virus; RV, rhinovirus.

Over the 6-month study period, the number of individuals with at least 1 infection of any of the target viruses, RV, AdV, HCoV, RSV, PIV, HMPV, and influenza were 75 (93.8%), 62 (77.5%), 57 (71.3%), 58 (72.5%), 52 (65.0%), 37 (46.3%), 11 (13.8%), and 8 (10.0%), respectively. The corresponding number of symptomatic infections of those ever infected, by pathogen, was 46 (61.3%), 33 (53.2%), 26 (45.6%), 29 (50.0%), 25 (45.5%), 11 (29.7%), 2 (18.1%), and 4 (50.0%), respectively. RV, AdV, HCoV, and RSV were the most prevalent respiratory viruses. They were each found in all the 6 households, infecting at least 1 member and were taken forward as the prevalent targets for screening of the NPS collection from the remaining 41 households. The temporal infection profile for the 6 households showing positive samples for each member is shown in Figure 1.

### Viruses Detected From the Select Respiratory Virus Screen

All the 16928 NPS collections from the 47 households had infection data from the 7 prevalent respiratory targets (RV, AdV, HCoV [OC43, NL63, and 229E], and RSV [groups A and B]). Of the 16928 NPS tested, 4259 (25.2%) were positive for 1 or more of the selected respiratory virus targets. Of the virus positives, 3687 (86.6%) were single virus detections, 526 (12.4) were dual, and 45 (1.1%) were triple, while only 1 (0.02%) had 4 targets codetected. Virus-positive specimens had a higher probability of being associated with respiratory symptoms compared with virus-negative specimens (34.1% [1450/4259] vs 16.7% [2114/1266], respectively; χ^2^*P* < .0001). The detected viruses, in order of frequency, were RV (1780 [10.5%]), HCoV (1274 [7.5%]), AdV (1232 [7.3%]), and RSV (537 [3.2%]). Of the HCoVs detected, 627 (49.2%), 399 (31.3%), and 212 (16.6%) were single infections of OC43, NL63, and 229E, respectively, and 36 (2.8%) had mixed HCoV strains. For the RSV-positive specimens, 231 (43.0%) and 287 (53.4%) had RSV group A and B only, respectively, while 19 (3.5%) specimens had both. Of all the virus-positive NPS collections, 657 (36.9%), 407 (33.0%), 410 (32.2%), and 229 (42.6%) had symptomatic infections with RV, HCoV, AdV, and RSV, respectively.

**Figure 2.**
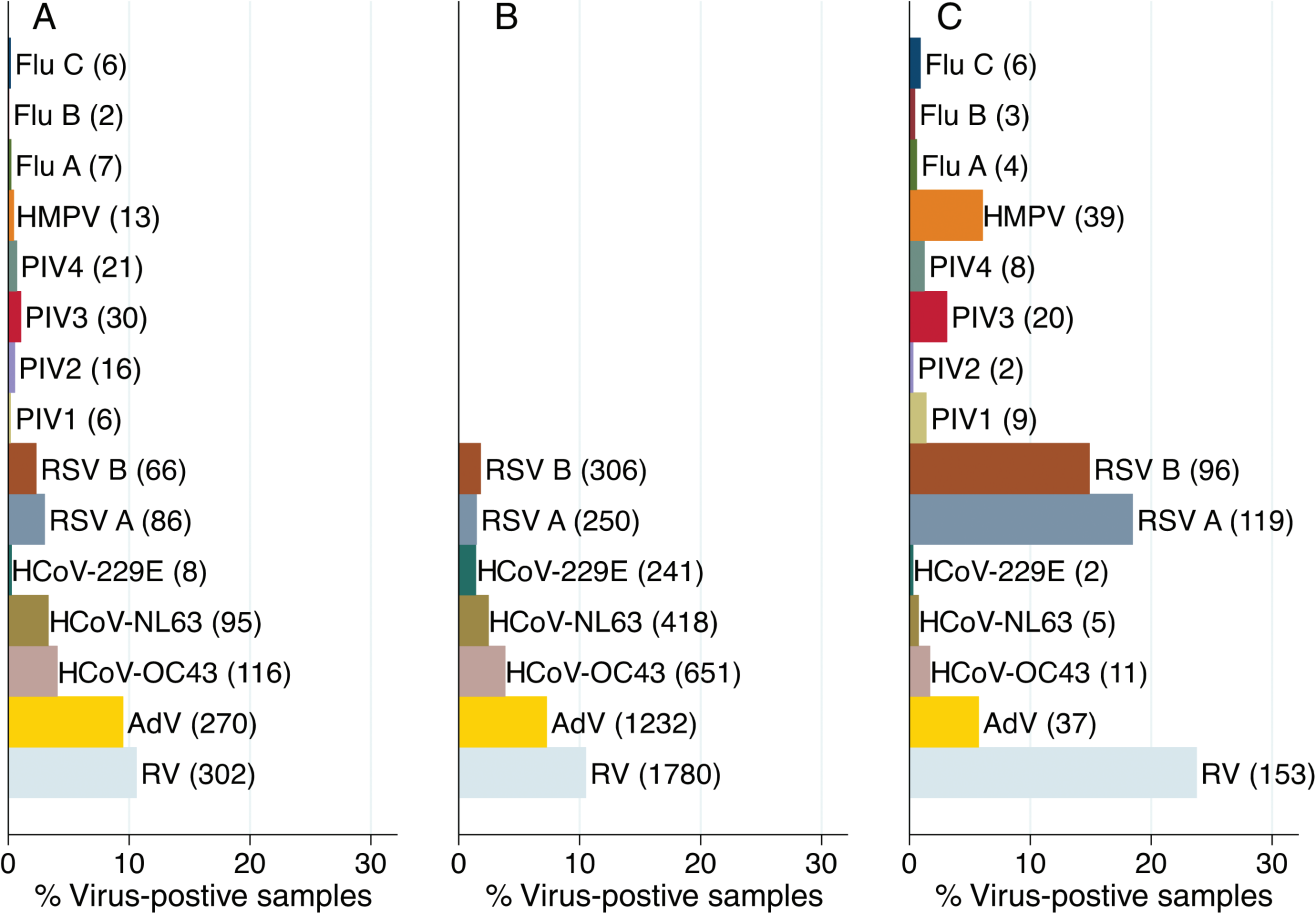
Frequency distribution of the detected respiratory viruses in NPS collections from the (*A*) six households with full respiratory screen and (*B*) the 47 households for the common targets screen and (*C*) inpatient samples collected over the same study period, December 2009–June 2010. Abbreviations: Adv, adenoviruses; Flu A, B, and C, influenza type A, B, and C; hCoV-OC43, hCoV-NL63 and hCoV-229E are strains of human coronaviruses; HMPV, human metapneumoviruses; NSP, nasopharyngeal swabs; PIV 1, 2, 3, and 4, parainfluenza type 1, 2, 3, and 4; RSV A and B, respiratory syncytial virus group A and B; RV, rhinoviruses.

The frequency distribution of the viruses circulating in the community during the study period are shown for the 6 households with full respiratory virus screen vs the 47 households with select respiratory screen in Figure 2A and 2B, respectively. For comparison, Figure 2C illustrates the frequency distribution of virus infections in pediatric (<5 years old) pneumonia admissions to Kilifi County Hospital over the same period [6], showing a markedly higher frequency of RV, RSV, HMPV, and PIV3 detections than in the households.

### Number of Different Respiratory Infections Over the Study Period

**Figure 3.**
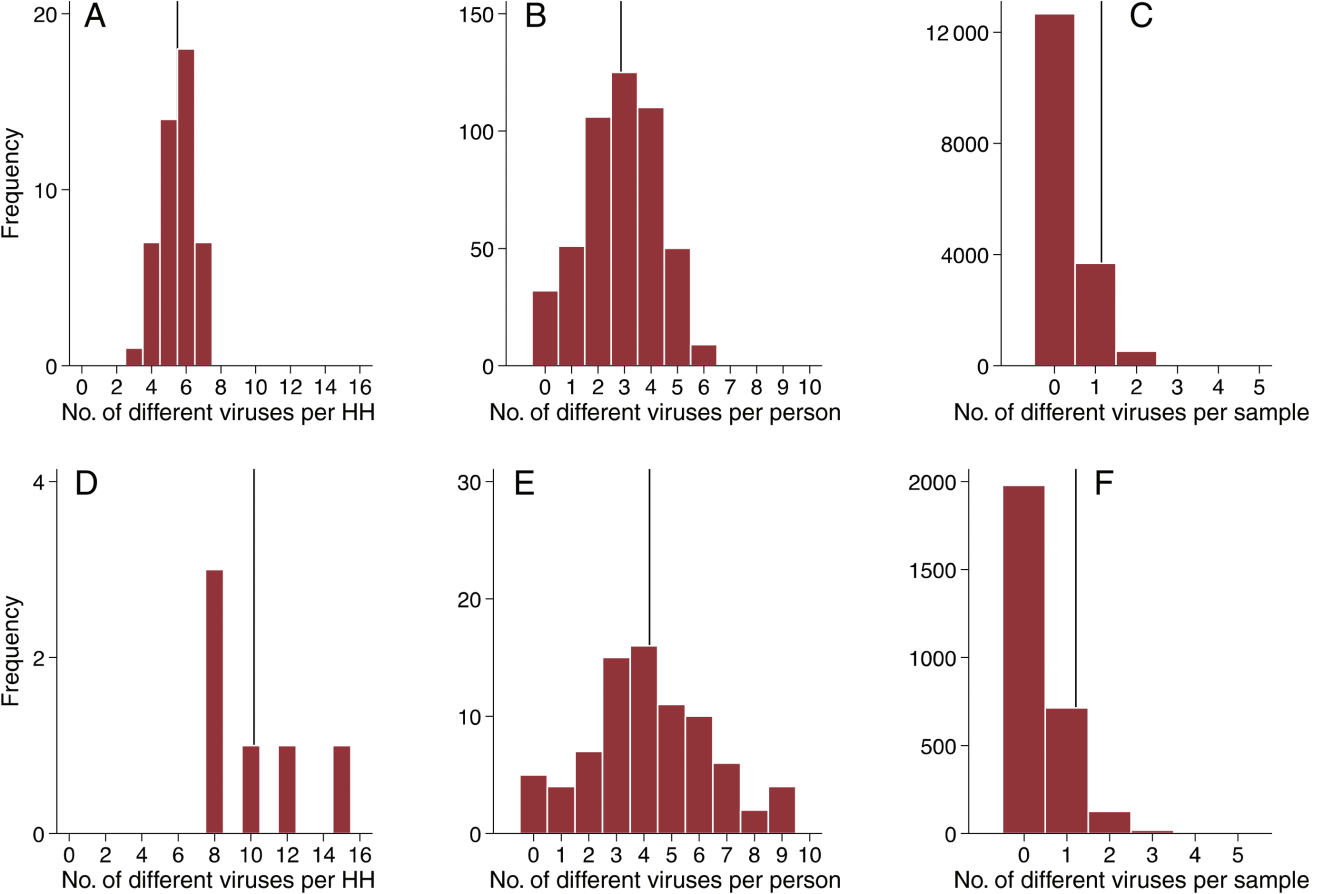
Frequency distribution of the number of different viruses detected per (*A* & *D*) household, (B & *E*) person and (*C* & *F*) per sample over the study period. Panels *A*–*C* represent the screening for common respiratory pathogens in all the 47 households while panels *D–F* show full screening in the six households. The vertical lines represent the respective mean values.

Of the 7 selected virus targets, each household had a median of 6 (range, 3–7) detected over the 6-month study period (Figure 3A). A higher median number (9 [range, 8–15]) of targets were detected for the 6 households with full respiratory virus screen (Figure 3D). At the individual level, a median of 3 different viruses (range, 0–6) were detected per person over the study period (Figure 3B). The corresponding median was 4 (range, 0–9) for the individuals with complete virus screening (Figure 3E). Of the virus-positive samples, 13.4% (572/4259) and 17.4% (150/864) had ≥2 viruses detected based on the screening of the select and full respiratory virus screen, respectively (Figure 3C and 3F).

### Seasonality of the Respiratory Viruses

**Figure 4.**
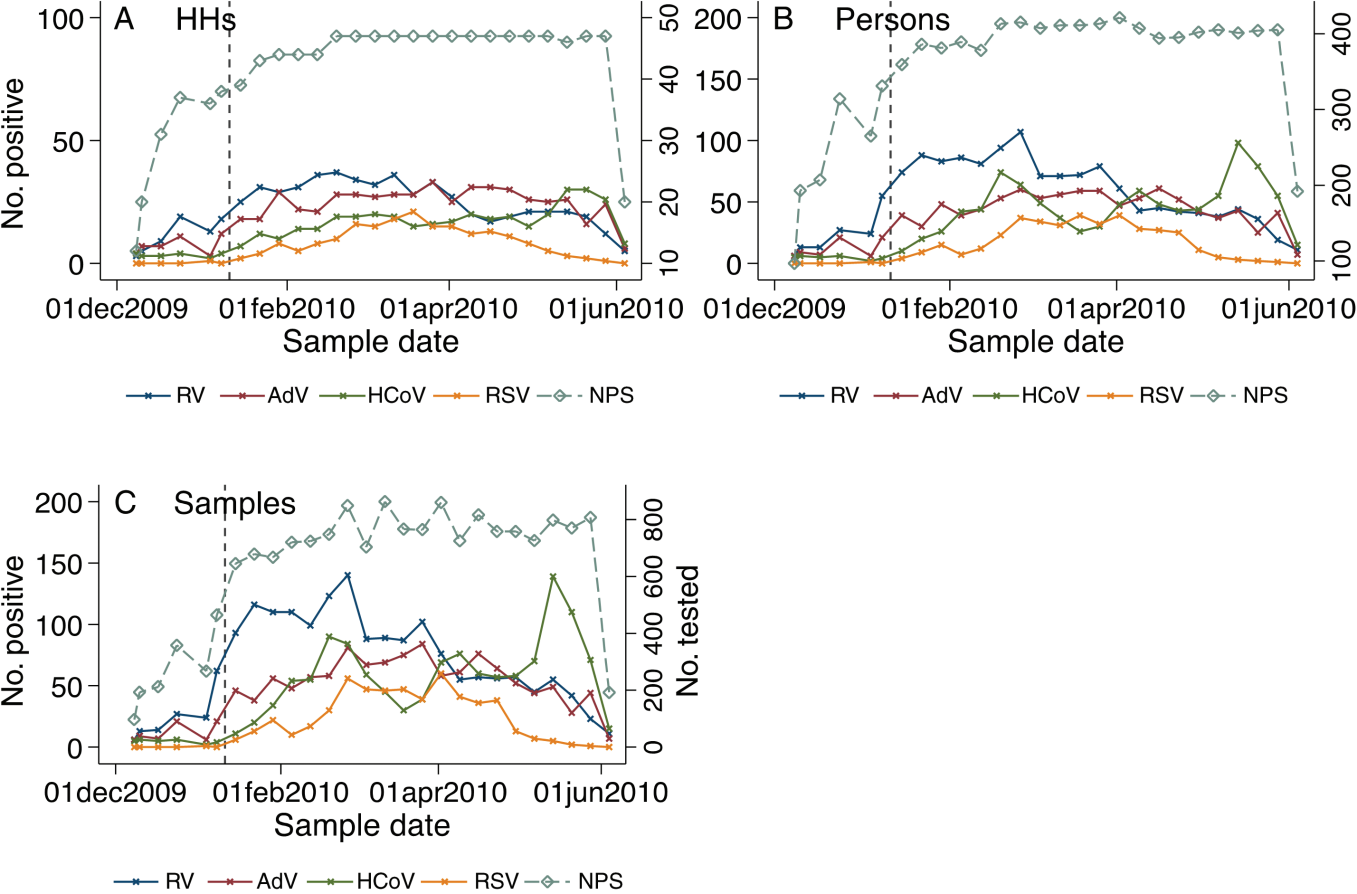
Number of nasopharyngeal swabs tested from the 47 households and viruses detected in households (*A*), persons (*B*), and samples per week (*C*) over the study period. The vertical dashed line denotes the start of the main study period, 10 January 2010. Abbreviations: AdV, adenovirus; HCoV, human coronavirus; HH, household; NPS, nasopharyngeal swab; RSV, respiratory syncytial virus; RV, rhinovirus.

RSV infections were first detected in the area from the hospital surveillance at the end of November 2009 (Supplementary Figure 1) but began circulating in the community study cohort in early January 2010 (Figure 4), peaking in March and fading out by the end of May 2010; the outbreak consisted of RSV A and RSV B at similar frequency (43.0% vs 53.4%). HCoV had 2 major peaks, 1 in February and 1 in May, and a minor peak in early April 2010. The major peaks were mainly linked with increased detection of both HCoV-OC43 and -NL63 while the minor peak was composed only of HCoV-OC43. Throughout the study, adenoviruses had a consistently high prevalence with no apparent peak times. The prevalence of RV was at its peak in January, gradually declined over the study period, and was at its lowest at the end of May 2010. The observed seasonal patterns were evident even after aggregating the data to assess the weekly detection rates of the viruses at sample, individual, or household level (Figure 4). From the 6 households with full respiratory virus screen, similar seasonal patterns were observed, albeit with greater variability (Supplementary Figure 2). The PIVs, influenza viruses, and HMPV were rarely detected throughout the 6-month study period (Supplementary Figure 2), and this was also observed from the hospital virus surveillance (Supplementary Figure 1).

### Household and Individual Risk of Infection Over the 6-Month Period

RV and HCoV were detected in all 47 households while AdV and RSV were detected in 45 (95.7%) and 40 (85.1%) households, respectively (Table 2). All of the households had at least 1 member with a symptomatic infection and symptomatic infections were detected in 46 of the households (97.9%) for RV, 45 (95.7%) for HCoV, 42 (89.4%) for AdV, and 34 (72.3%) for RSV. The individual risk of infection was 93.4% (451 individuals), 80.1% (387), 71.6% (346), 61.5% (297), and 37.1% (179) for any virus, RV, HCoV, AdV, and RSV, respectively (Table 2). The corresponding individual risk for symptomatic infections was 61.7% (298 individuals), 49.5% (239), 34.0% (164), 27.3% (132), and 22.0% (106), respectively.

### Individual Risk of Infection by Symptom Status

**Figure 5.**
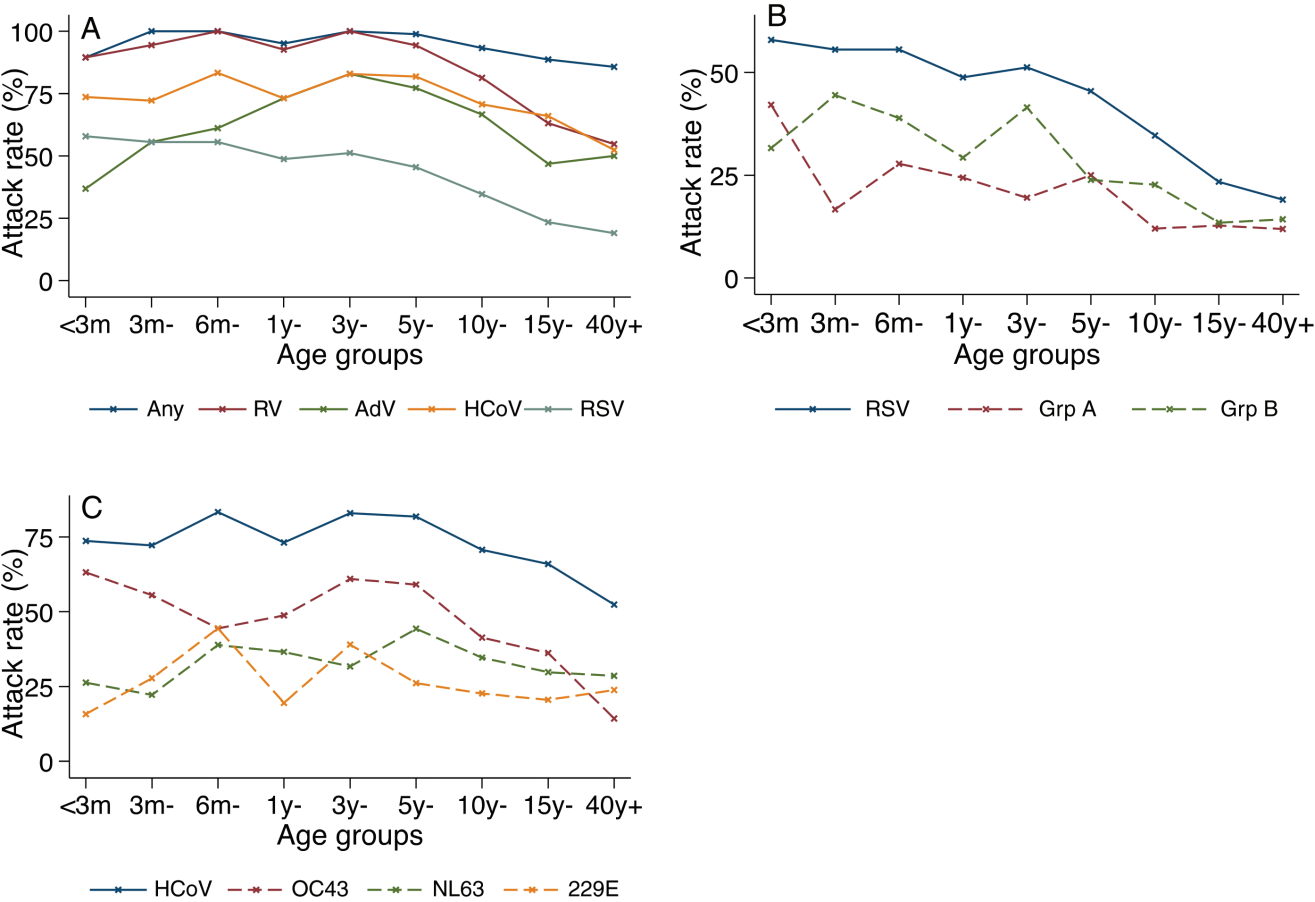
Age-specific attack rates for the common respiratory viruses (*A*), respiratory syncytial virus groups (*B*), and human coronavirus strains (*C*) among the 483 individuals sampled over the 6-month period. Abbreviations: AdV, adenovirus; HCoV, human coronavirus; RSV, respiratory syncytial virus; RV, rhinovirus.

Age-specific attack rates for the prevalent viruses significantly decreased with age (Table 3 and Figure 5). This age association was enhanced for the symptomatic infections. Unlike other targets whose the highest attack rates were among young children aged <1 year, AdV had highest attack rates among older children aged 1–4 years. The groupings based on age were closely related to those based on the relationship to the study infant; hence, the pattern of attack rates according to relationships was similar to that of the age groups (Table 3 and Supplementary Table 1). Notably, the attack rates regardless of symptoms in mothers were higher than in fathers for all the studied viruses, and this was significant statistically (*P* = .04). There were no statistically significant differences in the attack rates regardless of symptoms by sex and school-going status (Table 3). However, for the symptomatic infections the attack rates for RSV were significantly higher in males than in females (26.6% vs 18.2%; *P* = .026). The attack rates by household sizes varied by pathogen and illness status. Households with fewer household members (4–7 individuals) had higher attack rates than larger households that were statistically significant for RV, HCoV, and RSV irrespective of symptoms. For symptomatic infections, only RSV showed a significant association by household size (Supplementary Table 1).

**Table 3.** Crude Individual Attack Rates of the Common Respiratory Viral Infections Detected Regardless of Symptoms, Stratified by Various Characteristics

Characteristics	Category	No.	Any Virus		Rhinovirus		Adenovirus		Coronavirus		RSV	
Age, y	<1	55	53	**(96.4)**	52	**(94.5)**	28	**(50.9)**	42	**(76.4)**	31	**(56.4)**
	1–4	82	80	**(97.6)**	79	**(96.3)**	64	**(78.0)**	64	**(78.0)**	41	**(50.0)**
	5–14	163	157	**(96.3)**	144	**(88.3)**	118	**(72.4)**	125	**(76.7)**	66	**(40.5)**
	15–39	141	125	**(88.7)**	89	**(63.1)**	66	**(46.8)**	93	**(66.0)**	33	**(23.4)**
	≥40	42	36	**(85.7)**	23	**(54.8)**	21	**(50.0)**	22	**(52.4)**	8	**(19.0)**
Relation to the infant	The infant	47	46	**(97.9)**	45	**(95.7)**	26	**(55.3)**	37	(78.7)	27	**(57.4)**
	Sibling	162	157	**(96.9)**	154	**(95.1)**	124	**(76.5)**	124	(76.5)	87	**(53.7)**
	Cousin	124	116	**(93.5)**	100	**(80.6)**	76	**(61.3)**	91	(73.4)	56	**(45.2)**
	Mother	46	45	**(97.8)**	35	**(76.1)**	29	**(63.0)**	27	(58.7)	16	**(34.8)**
	Father	30	25	**(83.3)**	16	**(53.3)**	15	**(50.0)**	17	(56.7)	7	**(23.3)**
	Other HH member	74	62	**(83.9)**	37	**(50.0)**	27	**(36.5)**	50	(67.6)	22	**(29.7)**
Sex	Female	269	252	(93.7)	215	(79.9)	169	(62.8)	186	(69.1)	96	(35.7)
	Male	214	199	(93.0)	172	(80.4)	128	(59.8)	160	(74.8)	83	(38.8)
School-going	No	313	289	(92.3)	246	(78.6)	184	(58.8)	217	(69.3)	119	(38.0)
	Yes	170	162	(95.3)	141	(82.9)	113	(66.5)	129	(75.9)	60	(35.3)
No. of individuals per HH	4–7	95	93	(97.9)	84	**(88.4)**	66	**(69.5)**	75	**(78.9)**	48	**(50.5)**
	8–10	120	109	(90.8)	100	**(83.3)**	87	**(72.5)**	77	**(64.2)**	26	**(21.7)**
	11–16	144	135	(93.8)	110	**(76.4)**	77	**(53.5)**	97	**(67.4)**	49	**(34.0)**
	17–37	124	114	(91.9)	93	**(75.0)**	67	**(54.0)**	97	**(78.2)**	56	**(45.2)**

Data are presented as No. (%). The bold values indicate statistical significance based on χ^2^ test (*P* < .05).

Abbreviations: HH, household; RSV, respiratory syncytial virus.

## DISCUSSION

This is the first study to provide detailed infection patterns of respiratory viruses derived from a household-based active surveillance applying molecular techniques in low-income settings. Applying intensive sampling regardless of symptoms together with multiple virus diagnostics, our household study reveals a remarkably high prevalence of respiratory viruses in the rural setting of coastal Kenya. This demonstrates the extraordinarily enabling environment for virus spread coherent with earlier reports for RSV infections [5–7]. Although interpretation of virus presence by molecular diagnostics should be undertaken with care, it seems very plausible that households with young infants provide a reservoir of respiratory pathogens that are disseminated into the community.

Even though the study was designed to coincide with the local RSV season, a diverse range of respiratory viruses were shown to co-circulate. AdV, HCoV, and RV were the most prevalent during the RSV epidemic. These respiratory viruses were detected in a quarter of the tested samples. Similar circulation of respiratory viruses was observed from virus watch family studies (1960s–1970s) in Michigan and Seattle, Washington, despite using less-sensitive diagnostic techniques (culture and serology) [11–15]. In the US families, RV predominated after school opening, partly explaining the concordance findings as our surveillance covered school periods [10, 11, 15]. The Tecumseh family study identified OC43 as the most common HCoV strain, as was observed in the current study [15]. A recent US family study using molecular techniques identified NL63 as the most prevalent [16].

Some of the pathogens were uncommon, and it is likely that a seasonal peak of some viruses fell outside the study period. In this location, peak occurrence of influenza (A or B) is in the second half of each year based on inpatient pediatric surveillance [17, 18]. HMPV circulated prior to the start of RSV season, as shown from corresponding hospital data (Supplementary Figure 1), unlike previous studies reporting co-circulation with RSV [19].

Dual or multiple infections were common (range, 13.4%–17.4%). A prospective cohort study in a daycare center in the United States using comparable molecular techniques reported a coinfection rate of 27% among symptomatic young children [20], indicating that this high burden of viral coinfection, especially among children, is global. Detection of coinfection was higher among the symptomatic cases, as has been reported in hospital-based surveillance studies [21–23].

At least 1 virus was detected in 93.4% of the study participants over the 6-month study, and on average, each individual had evidence of 3 different viral infections. Given the close contacts of individuals in the households, the participants’ exposure to the investigated respiratory viruses was high: >95% of the households had 1 or more members detected with RV, HCoV, and AdV. The individual attack rates declined with increasing age for most of the target pathogens, most likely due to acquisition of immunity following previous infections. School-going children are usually respiratory virus introducers to households [6, 24], but did not seem to have higher individual attack rates compared to non-school-goers for the studied viruses. Fathers had consistently and significantly lower attack rates compared with mothers. In this community, fathers are likely to have fewer interactions with the young infants and children relative to mothers, which could partly explain the disparity in attack rates. Empirical data on contact patterns within households might help elucidate this observation. Individuals in larger households (>7 members) had lower attack rates than in smaller households, and this pattern was significant for RV, AdV, and RSV. This may be related to the structure of households, which comprise 1 or more building units, and larger occupancy would tend to have more building units, between which there may be less interaction than in a single-building household.

The frequency distribution of viruses in the community does not reflect that in the hospital, which provides a reminder that hospital data do not well describe infection transmission in the community, but rather the disease that arises, and this is clearly virus specific—that is, very much higher prevalence of RSV, HMPV, PIV3, and, interestingly, rhinoviruses among hospital cases than in the community.

The study has some limitations. First, the study was designed with a focus on RSV, and here we are presenting an observational data set from essentially a “convenience” sample for a small number of viruses for a short period. A surveillance over a longer period and investigating a wider range of respiratory viruses would provide more comprehensive data on virus circulation. Multiple years of study would compensate for year-to-year variation. Second, our sample was households with infants, so it might be possible that households without infants would have a lower prevalence. Third, only a small number of households had their samples subjected to full respiratory screen. Given the clustering of respiratory infections by households, it is possible that a different set of households might have resulted in an additional choice of targets for screening. However, the similarities in circulation of the respiratory viruses in the community study and the hospital surveillance do not support this view. Last, virus infection was deduced from molecular diagnostics, which do not necessarily equate with the presence of potentially infectious virus, leading to overestimation of infectiousness. Each multiplex in the molecular screen may have reduced sensitivity for detecting coinfections as compared to single-target assays.

In conclusion, respiratory virus infections and associated illness in this setting are ubiquitous in households. The molecular screen of these specimens revealed continuous and considerable respiratory virus circulation and infection frequency in this population that varied with virus species and subject age. The study here unveils previously unknown patterns of respiratory pathogen circulation in a rural low-income population. The remarkable frequency of virus infections of multiple species and strains lends itself to an ecological analysis of interactions that may be influential in virus ecology. The etiology of respiratory disease and immunological burden of respiratory viruses in children is worthy of further study. In addition, investigation on the human virome in the nasopharynx would provide insight on these viruses and how they affect human health and disease. Future studies should address the health and economic implications of these observations.

## Supplementary Data

Supplementary materials are available at *Clinical Infectious Diseases* online. Consisting of data provided by the authors to benefit the reader, the posted materials are not copyedited and are the sole responsibility of the authors, so questions or comments should be addressed to the corresponding author.

Supplementary Table S1

Supplementary Figure S1

Supplementary Figure S2

Supplementary Figure S3

Supplementary Figures Legend

## Notes

***Acknowledgments.*** We acknowledge the Viral Epidemiology and Control group at the Kenya Medical Research Institute (KEMRI)–Wellcome Trust Research Programme in Kilifi, Kenya, for their critique of an earlier version of the manuscript. Special appreciation goes to the study field workers and laboratory technicians for their commitment in the sample collection and testing, respectively. The article is published with the permission of the Director of KEMRI.

***Financial support.*** This work was supported by the Wellcome Trust, United Kingdom (grant numbers 102975, 090853, 084633, and 206748).

***Potential conflicts of interest.*** All authors: No potential conflicts. All authors have submitted the ICMJE Form for Disclosure of Potential Conflicts of Interest. Conflicts that the editors consider relevant to the content of the manuscript have been disclosed.
