# Supplementary material for: Continuous Invasion by Respiratory Viruses Observed in Rural Households During a Respiratory Syncytial Virus Seasonal Outbreak in Coastal Kenya
Source: Clin Infect Dis. 2018 Apr 16;67(10):1559–67. doi: 10.1093/cid/ciy313 (PMC6206121; doi:10.1093/cid/ciy313)
Supplement: Supplementary Table S1 [file ciy313_suppl_supplementary_table_s1.docx]

Table S1: Crude individual attack rates for the symptomatic respiratory viral infections stratified by various characteristics

| *Characteristics* |  | **N** | **Any virus** | | **Rhinovirus** | | **Adenovirus** | | **Coronavirus** | | **RSV** |  |
| --- | --- | --- | --- | --- | --- | --- | --- | --- | --- | --- | --- | --- |
|  | *Categories* | *N* | *n* | *%* | *n* | *%* | *n* | *%* | *n* | *%* | *n* | *%* |
| Age in years | <1 | 55 | 50 | **90.9** | 47 | **85.5** | 21 | **38.2** | 28 | **60.0** | 33 | **50.9** |
|  | 1-4 | 82 | 77 | **93.9** | 66 | **80.5** | 53 | **64.6** | 35 | **57.3** | 47 | **42.7** |
|  | 5-14 | 163 | 116 | **71.2** | 88 | **54.0** | 48 | **29.4** | 34 | **42.3** | 69 | **20.9** |
|  | 15-39 | 141 | 46 | **32.6** | 31 | **22.0** | 8 | **5.7** | 7 | **7.8** | 11 | **5.0** |
|  | ≥40 | 42 | 9 | **21.4** | 7 | **16.7** | 2 | **4.8** | 2 | **9.5** | 4 | **4.8** |
| Relation to the infant | The infant | 47 | 46 | **97.9** | 43 | **91.5** | 20 | **42.6** | 27 | **66.0** | 31 | **57.4** |
|  | Sibling | 162 | 130 | **80.2** | 118 | **72.8** | 70 | **43.2** | 44 | **46.3** | 75 | **27.2** |
|  | Cousin | 124 | 80 | **64.5** | 51 | **41.1** | 34 | **27.4** | 29 | **36.3** | 45 | **23.4** |
|  | Mother | 46 | 17 | **37.0** | 11 | **23.9** | 5 | **10.9** | 3 | **8.7** | 4 | **6.5** |
|  | Father | 30 | 7 | **23.3** | 2 | **6.7** | 1 | **3.3** | 2 | **10.0** | 3 | **6.7** |
|  | Other HH members | 74 | 18 | **24.3** | 14 | **18.9** | 2 | **2.7** | 1 | **8.1** | 6 | **1.4** |
| Sex | Female | 269 | 158 | 58.7 | 133 | 49.4 | 69 | 25.7 | 49 | 30.5 | 82 | **18.2** |
|  | Male | 214 | 140 | 65.4 | 106 | 49.5 | 63 | 29.4 | 57 | 38.3 | 82 | **26.6** |
| School going | No | 313 | 195 | 62.3 | 162 | 51.8 | 97 | **31.0** | 80 | 36.4 | 114 | **25.6** |
|  | Yes | 170 | 103 | 60.6 | 77 | 45.3 | 35 | **20.6** | 26 | 29.4 | 50 | **15.3** |
| Number of individuals per HH (household sizes) | 4 to 7 | 95 | 67 | 70.5 | 55 | 57.9 | 31 | 32.6 | 35 | 41.1 | 39 | **36.8** |
|  | 8 to10 | 120 | 77 | 64.2 | 66 | 55.0 | 38 | 31.7 | 15 | 34.2 | 41 | **12.5** |
|  | 11 to 16 | 144 | 80 | 55.6 | 65 | 45.1 | 31 | 21.5 | 29 | 29.9 | 43 | **20.1** |
|  | 17 to 37 | 124 | 74 | 59.7 | 53 | 42.7 | 32 | 25.8 | 27 | 33.1 | 41 | **21.8** |

Key: *Key: The bold values indicate statistically significant based on Chi-square tests, p-value<0.05.*
