# Supplementary figures and images for: Continuous Invasion by Respiratory Viruses Observed in Rural Households During a Respiratory Syncytial Virus Seasonal Outbreak in Coastal Kenya
Source: Clin Infect Dis. 2018 Apr 16;67(10):1559–67. doi: 10.1093/cid/ciy313 (PMC6206121; doi:10.1093/cid/ciy313)

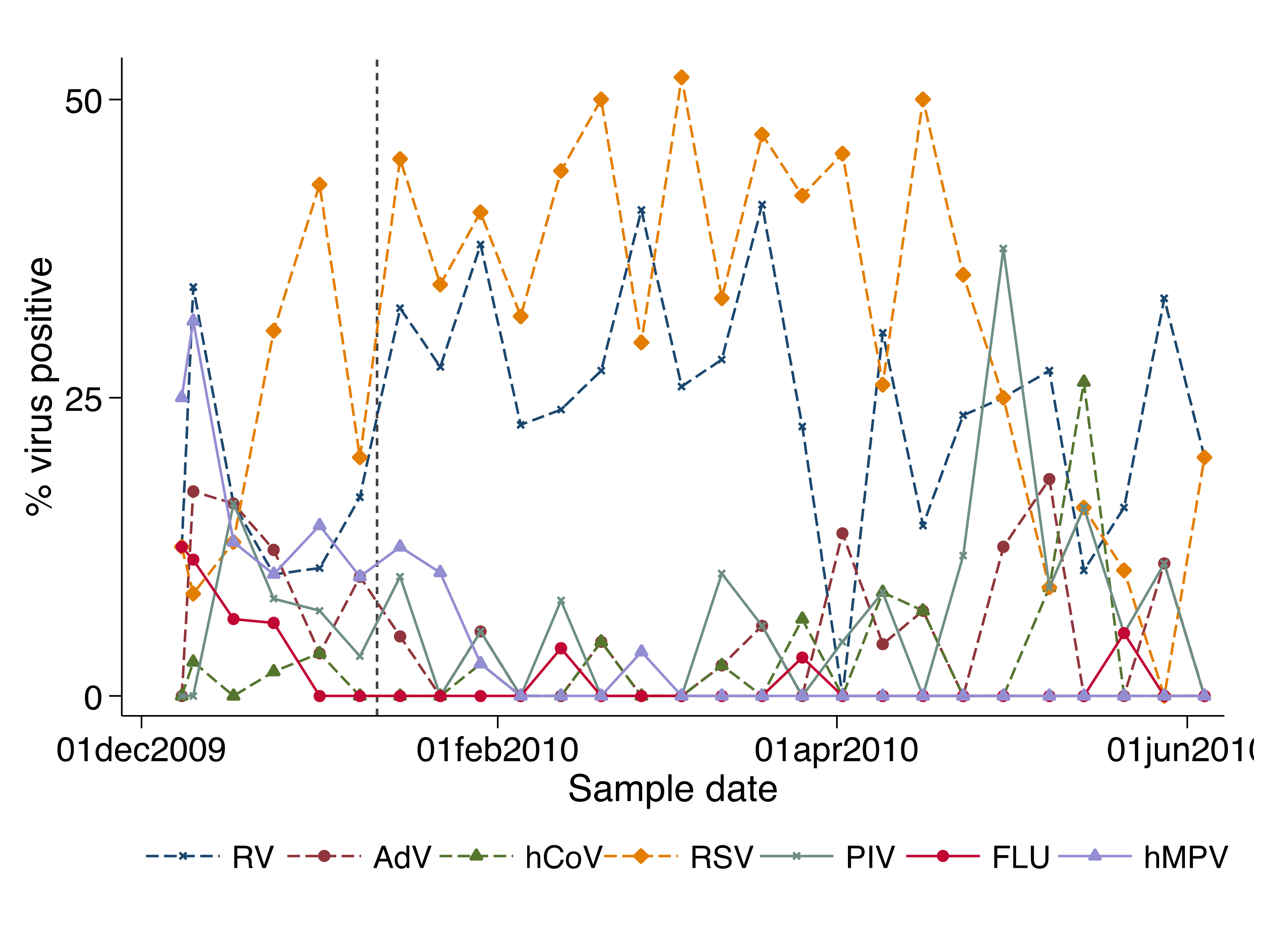

Supplement: Supplementary Figure S1 [file ciy313_suppl_supplementary_figure_s1.png]

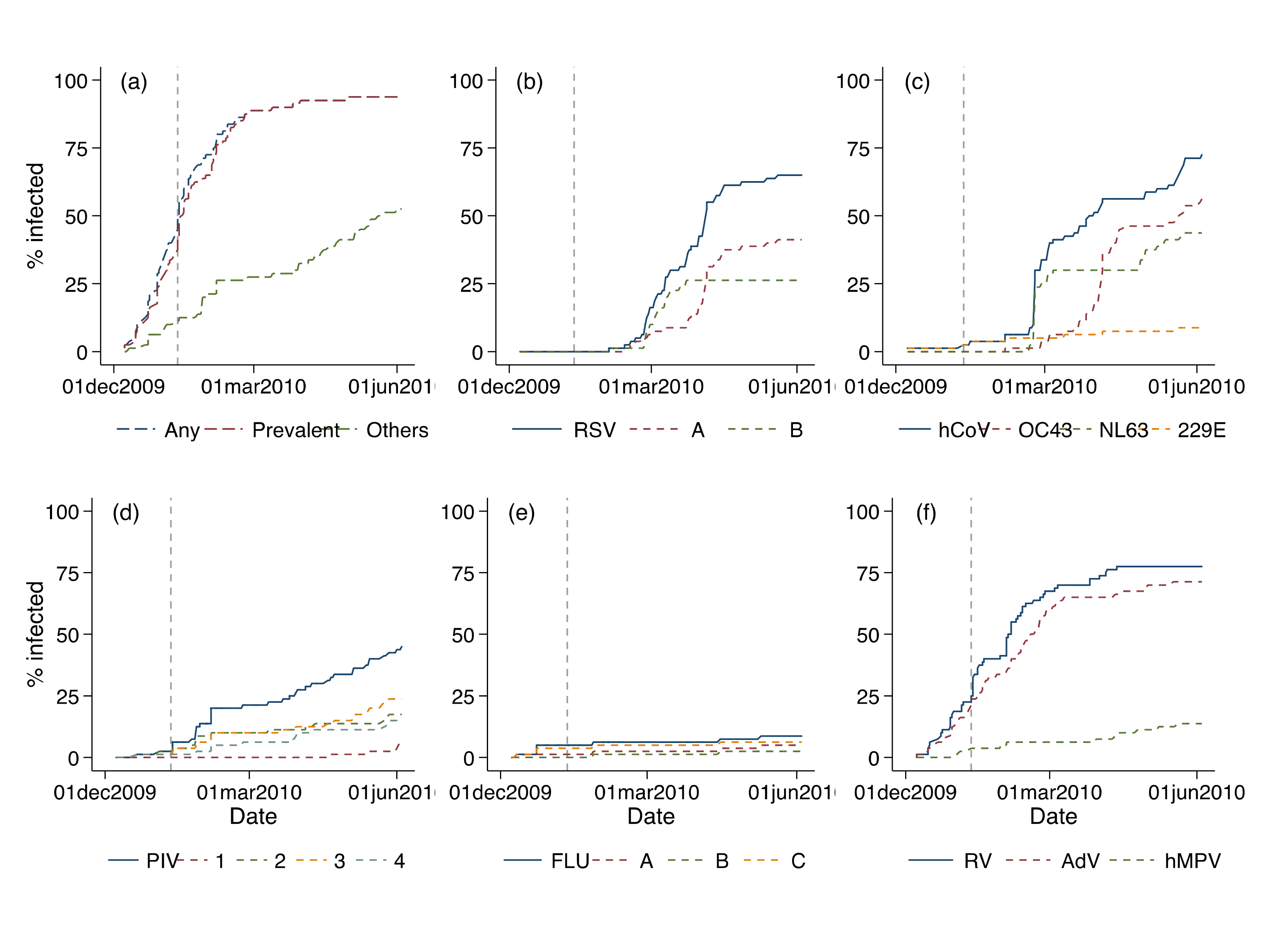

Supplement: Supplementary Figure S2 [file ciy313_suppl_supplementary_figure_s2.png]

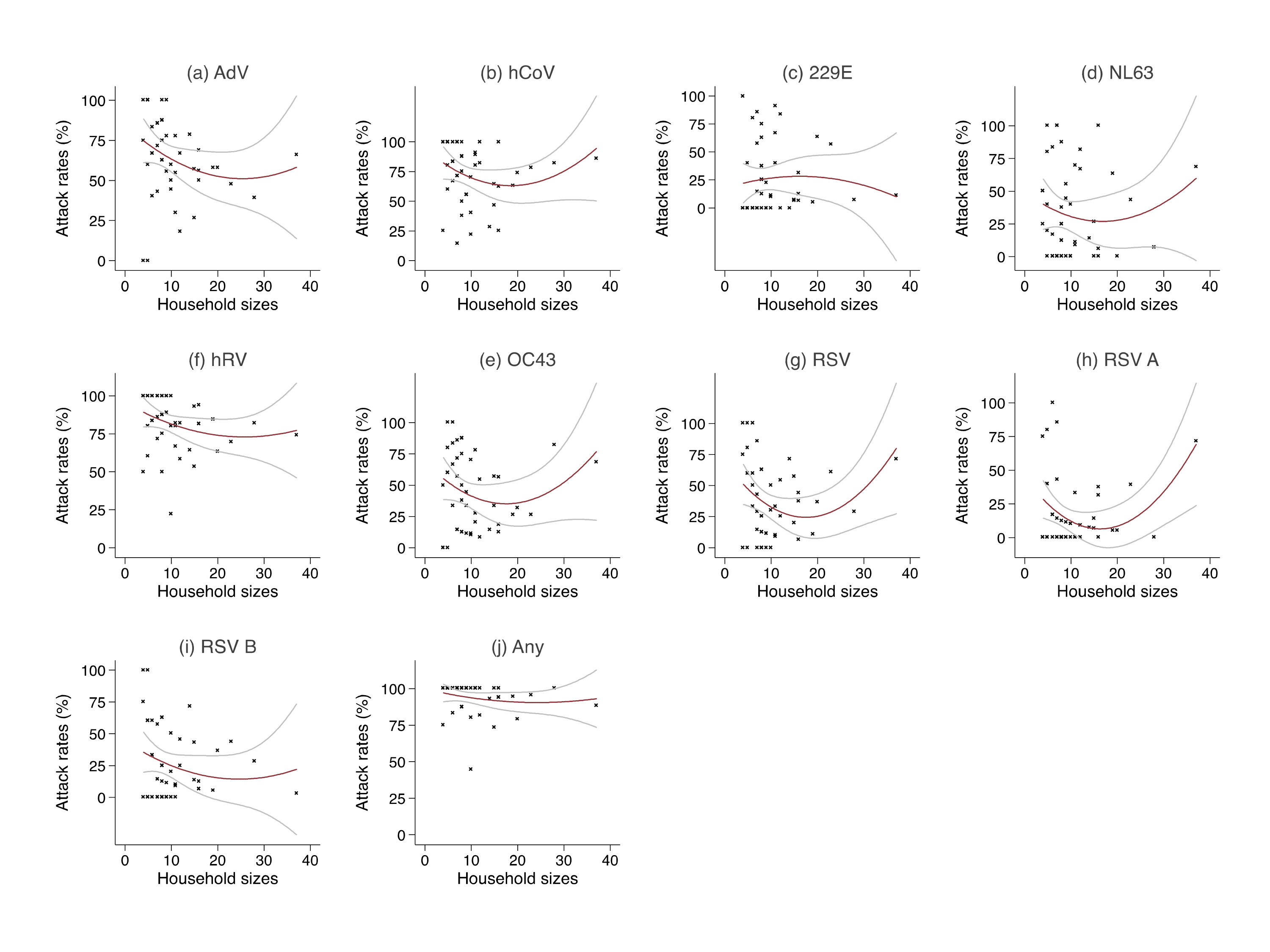

Supplement: Supplementary Figure S3 [file ciy313_suppl_supplementary_figure_s3.png]
