# Supplementary material for: Continuous Invasion by Respiratory Viruses Observed in Rural Households During a Respiratory Syncytial Virus Seasonal Outbreak in Coastal Kenya
Source: Clin Infect Dis. 2018 Apr 16;67(10):1559–67. doi: 10.1093/cid/ciy313 (PMC6206121; doi:10.1093/cid/ciy313)
Supplement: Supplementary Figures Legend [file ciy313_suppl_supplementary_figures_legend.docx]

**List of supplementary figures**

**Figure S1:** Prevalence of virus detection per week over the study period based on inpatient paediatric (<5 years) respiratory surveillance at Kilifi County Hospital. The vertical line shows the start of the main community study period, 10th Jan 2010.

**Figure S2:** Cumulative individual infection rates by (a) respiratory pathogens (b) RSV (c) hCoV, (d) PIVs, (e) Influenza viruses and (f) RV, AdV and hMPV from the six households with full respirartory screen

**Figure S3:** Percentage of individuals infected with (a) adenovirus, (b) human coronaviruses, (c) hCoV-229E, (d) hCoV-NL63, (e) hRV, (f) hCoV, (g) RSV, (h) RSV group A, (i) RSV group B and (j) any respiratory viruses per household by household size over the study period. The quadratic fit in red line and 95% CI in grey line.
